# Complex Enterally Tube-Fed Community Patients Display Stable Tolerance, Improved Compliance and Better Achieve Energy and Protein Targets with a High-Energy, High-Protein Peptide-Based Enteral Tube Feed: Results from a Multi-Centre Pilot Study

**DOI:** 10.3390/nu12113538

**Published:** 2020-11-18

**Authors:** Benjamin Green, Katy Sorensen, Mary Phillips, Lisa Green, Rachel Watson, Adrienne McCallum, Sarah Brook, Siobhan Oldham, Michelle Barry, Lyndsey Tomlinson, Alice Williams, Sam Crease, Carrie Wills, Rose Talbot, Rourke Thomas, Julie Barker, Annalisa Owen, Judith Davies, Carys Robinson, Anna Lumsdon, Samm Morris, Chloé McMurray, Nicola Cunningham, Lily Miller, Carolyn Day, Kristina Stanley, Susan Price, Susan Duff, Anna Julian, Jennifer Thomas, Carole-Anne Fleming, Gary Hubbard, Rebecca Stratton

**Affiliations:** 1 Medical Affairs, Nutricia, Trowbridge BA14 0XQ, UK; katy.sorensen@nutricia.com (K.S.); Gary.hubbard@nutricia.com (G.H.); Rebecca.stratton@nutricia.com (R.S.); 2 Royal Surrey County Hospital NHS Foundation Trust, Guildford GU2 7XX, UK; mary.phillips1@nhs.net; 3 Calderdale and Huddersfield NHS Foundation Trust, Huddersfield HD3 3EA, UK; Lisa.Green@cht.nhs.uk (L.G.); Rachel.Watson@cht.nhs.uk (R.W.); adrienne.mccallum@york.nhs.uk (A.M.); Sarah.Brook@cht.nhs.uk (S.B.); 4 Gloucestershire Hospitals NHS Foundation Trust, Gloucestershire GL53 7AN, UK; siobhan.oldham@nhs.net (S.O.); michelle.barry@nhs.net (M.B.); lyndsey.tomlinson@nhs.net (L.T.); alice.williams21@nhs.net (A.W.); 5 Somerset NHS Foundation Trust, Somerset TA6 4RN, UK; Sam.Crease@SomersetFT.nhs.uk (S.C.); Carrie.Wills@SomersetFT.nhs.uk (C.W.); Rose.Talbot@SomersetFT.nhs.uk (R.T.); 6 University Hospitals Bristol and Weston NHS Foundation Trust, Bristol BS1 3NU, UK; Rourke.Thomas@UHBW.nhs.uk (R.T.); Julie.Barker2@UHBW.nhs.uk (J.B.); 7 Aneurin Bevan University Health Board, Newport NP18 3XQ, UK; Annalisa.Owen@wales.nhs.uk (A.O.); Judith.Davies6@wales.nhs.uk (J.D.); 8 North Tees and Hartlepool NHS Foundation Trust, Stockton on Tees TS19 8PE, UK; Carys.robinson10@nhs.uk (C.R.); Anna.Lumsdon@nhs.net (A.L.); Samantha.Morris11@nhs.net (S.M.); Chloe.McMurray1@nhs.net (C.M.); 9 Northumbria Healthcare NHS Foundation Trust, North Shields NE29 8NH, UK; Nicola.Cunningham@northumbria-healthcare.nhs.uk (N.C.); Lily.Miller@northumbria-healthcare.nhs.uk (L.M.); 10 University Hospitals of Derby and Burton NHS Foundation Trust, Derby DE22 3NE, UK; carolyn.day1@nhs.net (C.D.); kristina.stanley@nhs.net (K.S.); 11 University Hospitals Birmingham NHS Foundation Trust, Birmingham B15 2GW, UK; Susan.Price@uhb.nhs.uk (S.P.); Susan.Duff@uhb.nhs.uk (S.D.); 12 NHS Greater Glasgow and Clyde, Glasgow G12 0XH, UK; Anna.Julian@ggc.scot.nhs.uk (A.J.); Jennifer.Thomas@ggc.scot.nhs.uk (J.T.); CaroleAnne.Fleming@ggc.scot.nhs.uk (C.-A.F.); 13 Faculty of Medicine, University of Southampton, Southampton SO17 1BJ, UK

**Keywords:** enteral nutrition, gastrointestinal tolerance, peptide, energy, protein, compliance

## Abstract

This pilot study evaluated a high-energy, high-protein, peptide-based, (medium-chain triglycerides) MCT-containing enteral tube feed (Nutrison Peptisorb Plus HEHP^®^, Nutricia Ltd., Trowbridge, BA14 0XQ, UK.) containing 1.5 kcal/mL and 7.5 g protein/100 mL. Fifteen community-based, enterally tube-fed adults (42 (SD 16.3) years) received the intervention feed daily for 28 days, with gastrointestinal tolerance, compliance and nutrient intake assessed at baseline and after the intervention period. Incidence and intensity of constipation (*p* = 0.496), nausea (*p* = 1.000), abdominal pain (*p* = 0.366) and bloating (*p* = 0.250) remained statistically unchanged, yet the incidence and intensity of diarrhoea improved significantly after receiving the intervention feed (Z = −2.271, *p* = 0.023). Compliance with the intervention feed was significantly greater compared to the patient’s baseline regimens (99% vs. 87%, *p* = 0.038). Compared to baseline, use of the intervention feed enabled patients to significantly increase total energy (1676 kcal/day (SD 449) to 1884 kcal/day (SD 537), *p* = 0.039) and protein intake (73 g/day (SD 17) to 89 g/day (SD 23), *p* = 0.001), allowing patients to better achieve energy (from 88% to 99%, *p* = 0.038) and protein (from 101% to 121%, *p* < 0.001) requirements. This pilot study demonstrates that a high-energy, high-protein, peptide-based, MCT-containing enteral tube feed maintains gastrointestinal tolerance and improves compliance, energy and protein intake in complex, enterally tube-fed, community-based adult patients, though more work is recommended to confirm this.

## 1. Introduction

Enteral tube feeding is a valuable method of nutritional support to ensure safe and sufficient delivery of nutrition directly to the gastrointestinal tract. Frequently used in hospital and community settings, enteral tube feeding supports both acute and chronically ill patients where oral feeding is not possible or insufficient to meet nutritional requirements [1]. In the UK, it is estimated that over 23,000 adults receive community-based, long-term Home Enteral Nutrition (HEN) [2,3,4], and HEN incidence across Europe has been estimated to be between 62 and 457 new patients per million inhabitants per year [5,6,7].

Depending on the severity of disease, surgery or treatment and ability to consume food orally, enteral tube feeding can be used as the sole source of nutrition (often termed “nutritionally complete”) or supplementary to oral intake, to meet the entire or partial nutritional requirements of patients. This is particularly pertinent in various nutritionally vulnerable populations at risk of disease-related malnutrition where oral feeding is contraindicated and nutritional requirements are elevated (namely energy and protein) [8,9]. The causes of disease-related malnutrition are multifactorial [8] and can include severe maldigestion and malabsorption of nutrients (including protein and fat), reduced gut motility, inflammation of the gut that leads to poor appetite, metabolic stress with high nutrient needs and an inability to take or tolerate oral feeding or some enteral tube feeds [10]. Intolerance to enteral tube feeding with polymeric whole protein and long-chain triglyceride fat-containing feeds is common, occurring in approximately 27–60% [11,12] and 30–40% [although anecdotal] of patients in critical care and community settings, respectively. Intolerance issues are likely a consequence of impaired feed assimilation due to inflammation, insufficient secretion of pancreatic enzymes or bile and/or reduced surface area for nutrient absorption, and patients commonly present with nausea, diarrhoea and abdominal distension [13]. If improperly managed, reduced nutritional intake and absorption or increased nutritional losses can occur, causing difficulties in meeting energy and protein goals [14], which ultimately augments the risk of disease-related malnutrition.

Provision of enteral tube feeds that contain hydrolysed, peptide-based proteins and readily absorbed fat such as medium-chain triglycerides (MCT) can help mitigate gastrointestinal tolerance issues in patients with impaired gastrointestinal function, maldigestion and/or malabsorption [15,16,17,18]. Indeed, better tolerance, digestion and absorption with peptide-based enteral tube feeds has been shown among various patient groups including those with inflammatory bowel disease [19,20], pancreatitis [21,22], pancreatectomy [23], radiation enteritis and chemotherapy [24], HIV-related gastrointestinal (GI) disorders [25] and short bowel syndrome [17,26]. In pancreatitis patients, for example, peptide-based formulas have been shown to lead to improved outcomes, such as less weight loss and shorter hospital stay, compared to patients taking polymeric whole protein formulas [22]. Furthermore, patients with a short bowel had enhanced nitrogen absorption when a peptide-based formula was used compared with a polymeric whole protein formula [17,26]. In addition, as fat malabsorption is a frequent problem in patients with impaired gastrointestinal function, the use of MCTs in peptide-based enteral tube feeds has been shown to decrease steatorrhea, decrease dyspepsia and improve nutritional status [27,28]. Therefore, peptide-based, MCT-containing enteral feeds offer nutritional, functional and clinical benefits among various patient groups; however, much of the existing evidence is in adults in critical care or other acute hospital settings. Evidence in patients receiving peptide-based HEN in the community is sparse in adult patients [28], and there are only a few studies in infant and paediatric populations [29,30], so more work is necessary.

Commercially available peptide-based enteral tube feeds have historically provided 1 kcal/mL with a protein content of approximately 4 g/100 mL, being nutritionally complete in 1500 mL. In recent years, the recognition of the need for higher protein provision has resulted in the development of more energy-dense, high-protein formulations. More energy- and protein-dense formulations can reduce the need for large volumes to satisfy already elevated nutritional requirements, which may further amplify gastrointestinal tolerance issues [14]. To overcome volume-associated gastrointestinal issues, together with the need to meet increased nutritional requirements for energy and protein, enteral tube feeds with higher caloric and protein provision are recommended. The aim of this pilot study was therefore to evaluate gastrointestinal tolerance, compliance and nutrient intake to a high-energy, high-protein, peptide-based, MCT-containing enteral tube feed (Nutrison Peptisorb Plus HEHP^®^, Nutricia Ltd., Trowbridge, BA14 0XQ, UK., herein referred to as HEHP) containing 1.5 kcal/mL and 7.5 g protein/100 mL, over a 28-day intervention period in adult, community-based, enterally fed patients.

## 2. Materials and Methods

### 2.1. Recruitment and Study Population

Enterally tube-fed patients were screened and recruited from inpatient and community services by their managing dietitian across eleven hospitals in the UK. Adult, enterally tube-fed patients were eligible for inclusion if they were aged 18 years and over, required a peptide-based feed to meet nutritional requirements and had to receive a minimum of 500 kcal/day of the intervention feed. Written informed consent was obtained from the patient and/or carer or a completed consultee declaration form for adults lacking capacity. Patients were excluded if they were receiving total parenteral nutrition, presented with major hepatic dysfunction (i.e., decompensated liver disease), major renal dysfunction (i.e., requiring filtration or Stage 4/5 chronic kidney disease), were in intensive care, had galactosaemia or severe lactose intolerance or had participated in other clinical studies within 2 weeks prior to entry to this study. Patients had to complete a minimum of 14 days of the intervention, as per the protocol, to be included in the final analysis. Outside of the inclusion and exclusion criteria, it was the dietitian’s clinical judgement as to which patients were appropriate to invite to join the study regardless of baseline feed usage. Furthermore, at the time of recruitment, the recruiting dietitian assessed the patients’ willingness/ability to follow the trial protocol, which included their ability to describe their gastrointestinal symptoms appropriately.

### 2.2. Study Design and Ethics

This was a prospective, single-arm, multicentre pilot study. Community-based patients attended their respective hospital (or were visited in the community) on two occasions separated by 28 days, for review with their managing dietitian. Following a detailed baseline patient history, where baseline gastrointestinal tolerance, compliance and nutrient intake to usual feeding regimens were established, each recruited patient received the intervention feed daily for 28 days. Gastrointestinal tolerance, compliance and nutrient intake were established again at the end of the intervention period. The intervention feed was provided in 500 mL ready-to-feed packs and was labelled specifically for this study. The intervention feed was a multi-nutrient, high-energy (1.5 kcal/mL), high-protein (7.5 g protein/100 mL), peptide-based, MCT-containing, ready-to-use enteral tube feed, nutritionally complete in 1000 mL. It was based on 100% hydrolysed whey protein (96.2% < 3000 Da, of which 40.6% 500–1000 Da and 41.5% < 500 Da) with 20% of energy from protein, 51% of energy from carbohydrates and 29% from fat (60% as MCT). The appropriate feed prescription was determined on an individual basis by the dietitian responsible for the patient’s nutritional management, based on the patient’s clinical requirements and preference and the dietitian’s clinical judgement. The intervention feed could be given as either a sole source of nutrition or alongside oral food intake and/or other enteral tube feeds. Details of the nutritional composition of the intervention feed are provided in Table 1.

The UK National Health Service (NHS) Research Ethics Committee (South Central—Berkshire Research Ethics Committee; 16/SC/0651) and local NHS R&D departments reviewed the experimental procedures and approved the study. The study was conducted in accordance with the Declaration of Helsinki of 1975, as revised in 2013, and ICH Good Clinical Practice. All patients provided written informed consent before any study-related procedures were performed.

### 2.3. Gastrointestinal Tolerance

The incidence and intensity of gastrointestinal symptoms (diarrhoea, constipation, nausea, abdominal pain and bloating) were recorded by patients on a 4-point Likert scale (Absent, Mild, Moderate, Severe) at baseline based on patients’ regimen at recruitment and at the end of the intervention period.

### 2.4. Compliance with Baseline Tube Feeding Regimen and HEHP Regimen

Details about each patient’s feeding regimen at recruitment (as prescribed by the patient’s managing dietitian) were assessed against usual intake data provided by the patient to calculate baseline percentage compliance. Volume intake of the intervention feed (in mL) was reported daily by the patient or carer and compared against the daily volume which the dietitian had prescribed. Average percentage compliance was calculated for the intervention period.

### 2.5. Total Energy and Protein Intake

During recruitment, on a per patient and condition-specific basis, the patient’s managing dietitian calculated estimated energy and protein requirements using recognised disease-specific calculations. Intake of all nutrition provided (including the intervention feed, other enteral tube feeds, foods, drinks and oral nutritional supplements) was recorded at baseline and at the end of the intervention period via 24 h dietary recall. Actual intakes were compared against the dietitian’s calculated estimated energy and protein requirements where percentage achievements were calculated. The 24 h dietary recall was undertaken by the patients managing dietitian. Dietary data were analysed using nutritional software (Nutritics Academic Edition V4.312, Dublin, Ireland) to calculate actual energy and protein intake.

### 2.6. Anthropometry

Body weight (kg) was measured to the nearest 0.1 kg using a weighing scale, without shoes or heavy clothing, at baseline and on day 28. Height (m) was captured at baseline and used to calculate body mass index (BMI, kg/m^2^) at baseline and on day 28. Where standing height could not be measured, estimated height was taken from knee height, ulna length or segmental length [31].

### 2.7. Dietetic Goal

As an adjunct to the wider aim of this pilot study, reasons for recruitment and, consequently, dietetic goals varied across patients, which is reflective of clinical practice. Therefore, at baseline, all dietitians were requested to set individual dietetic goals for their patients. After 28 days of receiving the intervention feed, the dietetic goals were assessed, and dietitians reported whether goals had been achieved (via Yes/No responses).

### 2.8. Safety

Adverse and serious adverse events (SAEs) were recorded throughout the study to assess potential safety issues related to the intervention feed. All events were recorded on appropriate forms where information concerning the intensity and potential relatedness to the intervention feed was sought.

### 2.9. Statistical Analysis

As this was a pilot study, with gastrointestinal tolerance as the primary outcome, the complexity and lack of data from studies of similar feeds rendered a power calculation on this outcome difficult, yet this was comparable to studies in similar populations [32]. Statistical procedures were performed using software package IBM SPSS Statistics v24 (IBM SPSS v24.0, Armonk, NY, USA). Data were checked for normal distribution with the use of the Kolmogorov–Smirnov normality test and were log-transformed if appropriate before statistical analysis. For continuous data (compliance, nutrient intake and anthropometry data), paired samples t-tests were used for comparisons of two time points (baseline vs. day 28). For non-parametric data relating to changes over time (gastrointestinal tolerance), Wilcoxon tests were used. Analysis was also split based on patients’ baseline feeding regimen (existing peptide feed users (*n* = 11) or existing polymeric feed users (*n* = 4)). Statistical significance was accepted at an α level of *p* < 0.05. All data are presented as mean (SD) unless otherwise stated. Fifteen patients who completed a minimum of 14 days intervention as per the protocol were included in the final analysis.

## 3. Results

### 3.1. Recruitment and Patient Characteristics

**Figure 1:**
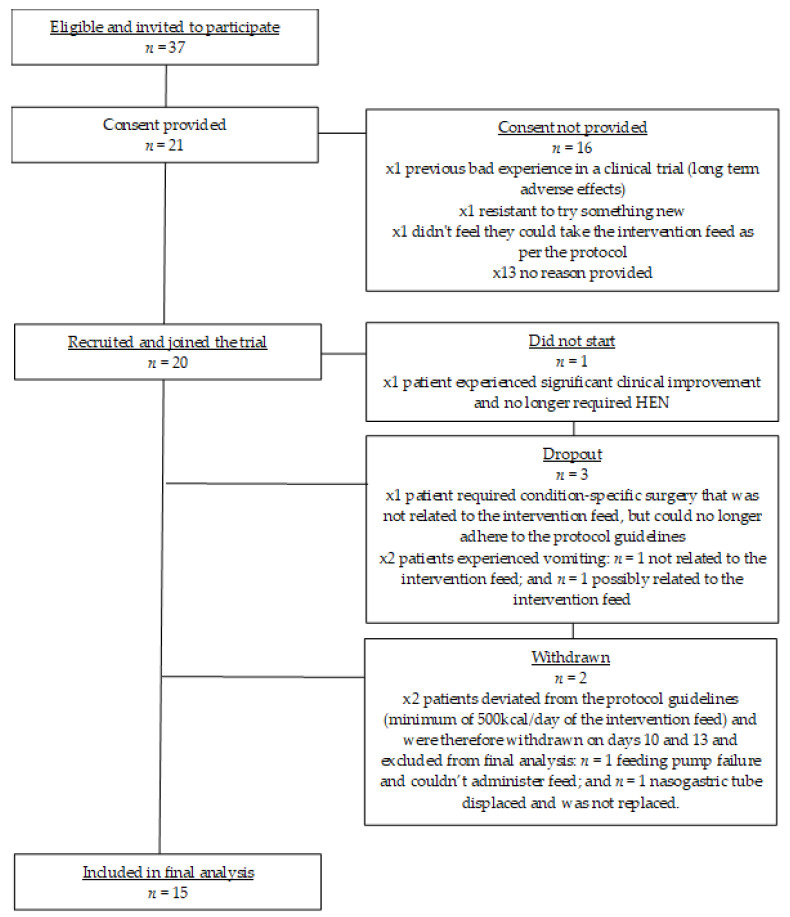
Flow chart of trial participation and exclusions.

See Figure 1 for a flow chart of trial participation and exclusions for this pilot study. From 11 UK study sites, 37 patients were invited to participate in this pilot study, of which 21 patients were assessed for eligibility and consented to take part. Due to significant clinical improvement, one patient no longer required enteral tube feeding and consequently did not start the study. From the 20 patients that joined the study and completed baseline measures, one dropped out before completing 7 days due a serious adverse event (patient required condition-specific surgery and could no longer adhere to the protocol guidelines) that was deemed unrelated to the intervention feed. Two patients withdrew due to vomiting and these adverse events were deemed unrelated (*n* = 1) and possibly related (*n* = 1) to the intervention feed. Two patients failed to meet the minimum daily intake of the intervention feed (protocol deviation) and were therefore excluded from final data analysis. One patient experienced a feeding pump failure and could therefore not administer the feed. The other patient’s nasogastric tube moved position and came out. The patient’s clinical team made the decision not to replace the feeding tube, so the patient had no means of taking the enteral feed. 

Characteristics of the 20 patients who were recruited and joined the trial are provided in Table 2 and Table 3.

Of the 15 patients who completed a minimum of 14 days intervention as per the protocol and were therefore included in the final analysis, patients had a mean age of 42 years (SD 16.3) (*n* = 8 females and *n* = 7 males) and a mean BMI of 22.5 kg/m^2^ (SD 5.1) and had estimated energy and protein requirements of 1914 kcal/d (SD 277) and 74 g/day (SD 17.9), respectively. All patients had complex conditions and 87% (*n* = 13) presented with multiple diagnoses. All patients were managed in the community and were recruited from home visits (*n* = 11) or during hospital admission for placement of an enteral feeding tube (*n* = 4), with most patients residing in their own homes (87%) and the remainder in care homes. All patients were receiving an enteral tube feed prior to recruitment and current feeding routes were maintained during this study. Patients were fed via a nasojejunal tube (*n* = 4), low profile balloon gastrostomy (*n* = 3), percutaneous endoscopic jejunostomy (*n* = 3), surgical jejunostomy (*n* = 30) and percutaneous endoscopic gastrostomy (*n* = 2). At baseline and during the study, 87% of patients (*n* = 13) were continuously feeding and 13% (*n* = 2) were bolus feeding. At baseline, *n* = 11 patients were receiving a 1–1.5 kcal/mL and 4–6.75 g protein/100 mL peptide-based enteral feed (Vital 1.5 kcal^®^, Abbott Nutrition, *n* = 7; Peptamen HN^®^, Nestlé Health Science, *n* = 2; Nutrison Peptisorb^®^, Nutricia Ltd., Trowbridge, BA14 0XQ, UK *n* = 2) primarily because of gastrointestinal tolerance issues (*n* = 7 of 11), but also to meet elevated nutrient needs (*n* = 2 of 11), need for a high nitrogen feed and low volume (*n* = 1 of 11) and pancreatic exocrine insufficiency (*n* = 4 of 11). The remaining patients (*n* = 4) were receiving a polymeric whole protein feed (Nutrison Energy^®^, Nutricia Ltd., *n* = 1; Nutrison Protein Plus^®^, Nutricia Ltd., Trowbridge, BA14 0XQ, UK *n* = 1; Nutrison Concentrated^®^, Nutricia Ltd., *n* = 1; Nutrison Energy Multi Fibre^®^, Nutricia Ltd., Trowbridge, BA14 0XQ, UK *n* = 1) to meet nutrient needs (*n* = 4 of 4). At baseline, 10 patients were nil by mouth (NBM), and five patients had some oral food intake established. The intervention feed was prescribed as the sole source of nutrition for 20% (*n* = 3) of patients, in addition to other enteral tube feeds for 20% (*n* = 3) of patients and in addition to oral intake for 60% (*n* = 9) of patients (either oral nutritional supplements (*n* = 4) or oral food intake (*n* = 5). The mean baseline feed prescription for those receiving a peptide-based feed was 980 mL/day (SD 453: range; 340–1760) or 1361 kcal/day (SD 625: range 500–2025) and for those receiving a polymeric feed was 1000 mL/day (SD 408: range 500–1500) or 1515 kcal/day (SD 568: range; 1000–2310). During the study, the mean prescription of the intervention feed for all patients was 871 mL/day (SD 396: range; 340–1500) or 1317 kcal/day (SD 604: range; 510–2250). When split according to baseline feeding regimen, the mean prescription of the intervention feed (824 mL (SD 400) or 1250 kcal (SD 617)) was significantly lower compared to baseline peptide-based enteral feed prescription (*p* = 0.050), yet not significantly lower compared to baseline polymeric enteral feed prescription (1000 mL (SD 408) or 1500 kcal (SD 612) *p* = 1.000) but this may be attributed to the small number of recruited polymeric users. For the duration of the trial, most patients (*n* = 11 of 15) were set multiple dietetic goals related to improved gastrointestinal tolerance, weight and nutritional status maintenance. Other goals included meeting nutrient requirements, reintroducing oral diet, increasing weight, tolerating increased volume of enteral feed and reducing the need for pancreatic enzyme replacement therapy (PERT).

### 3.2. Gastrointestinal Tolerance

**Figure 2:**
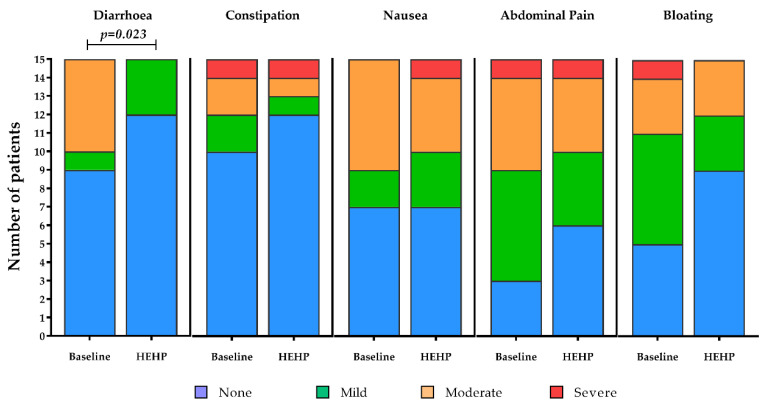
Incidence (number of patients) and intensity of diarrhoea, constipation, nausea, abdominal pain and bloating recorded on a 4-point Likert scale (Absent, Mild, Moderate, Severe) at baseline and with the intervention feed, as depicted by the HEHP bars.

The majority of gastrointestinal symptoms were absent at baseline and after receiving the intervention feed, where the incidence and intensity of constipation (*p* = 0.496, Figure 2), abdominal pain (*p* = 0.366, Figure 2), nausea (*p* = 1.000, Figure 2) and bloating (*p* = 0.250, Figure 2) remained statistically unchanged. The incidence and intensity of diarrhoea, however, improved significantly after receiving the intervention feed (Z = −2.271, *p* = 0.023, Figure 2). In this sense, improvements were seen in 40% of patients (*n* = 6 of 15), with symptoms unchanged in the remaining 60% of patients (*n* = 9 of 15, mostly remaining as “absent”).

For patients that were existing peptide feed users, similar results were observed, with symptom incidence and intensity reductions for constipation, nausea, abdominal pain and bloating with the intervention, but these did not reach statistical significance (*NS p* > 0.05 for all); however, incidence and intensity of diarrhoea significantly improved with the intervention feed compared to baseline (Z = −2.121, *p* = 0.034). For patients who were existing polymeric feed users, most gastrointestinal symptoms were absent and for others remained unchanged between baseline and after receiving the intervention feed. Some improvements in the intensity of symptoms were observed but, likely due to the small number of patients taking a polymeric feed at baseline, differences did not reach statistical significance (*NS p* > 0.05 for all).

### 3.3. Compliance with Baseline Tube Feeding Regimen and HEHP Regimen

Compliance with the intervention feed was excellent, with a mean compliance of 99% (SD 4) versus managing dietitian prescription. This was significantly greater than patients’ baseline regimen compliance (87% (SD 18); *p* = 0.038).

No statistically significant difference in compliance was, however, observed between baseline and with the intervention feed for existing peptide feed users (92% (SD 11) at baseline vs. 99% (SD 3) with the intervention feed, *p* = 0.086) or existing polymeric feed users (75% (SD 29) at baseline vs. 99% (SD 8) with the intervention feed, *p* = 0.233).

### 3.4. Total Energy and Protein Intake

Total energy intake (including the intervention feed, other enteral tube feeds, foods, drinks and oral nutritional supplements) increased significantly from 1675 kcal/day (SD 449) at baseline to 1884 kcal/day (SD 537) at the end of the study (*p* = 0.039, *n* = 15). Total energy intake as a percentage of estimated requirements also increased significantly from 88% (SD 30) at baseline to 99% (SD 25) at the end of the study (*p* = 0.038). Similarly, total protein intake (including the intervention feed, other enteral tube feeds, foods, drinks and oral nutritional supplements) increased significantly from 73 g/day (SD 17) at baseline to 89 g/day (SD 23) with the intervention feed (*p* = 0.001, *n* = 15). Total protein intake as a percentage of estimated requirements also increased significantly from 101% (SD 23) at baseline to 121% (SD 25) with the intervention feed (*p* < 0.001) (see Table 4).

During the study, clinical condition improved for several patients while taking the intervention feed, and *n* = 7 patients had some oral food intake established at the end of the study compared to *n* = 5 at baseline. Oral energy and protein intake in those patients with some established oral intake was 424 kcal/day (SD 288) and 22 g/day (SD 18.7) at baseline and 718 kcal/day ((SD 355) *p* = 0.144) and 30 g/day ((SD 18) *p* = 0.068) at the end of the study. The mean energy and protein intake from the intervention feed was 1237 kcal/day (SD 602) and 56 g/day (SD 26) and was comparable to mean energy and protein intake for the patients who at baseline were receiving a peptide-based enteral feed (1255 kcal/day (SD 593), *p* = 0.797 and 62 g/day (SD 30), *p* = 0.244 (*n* = 11 for both)). Nonetheless, total energy intake (including the intervention feed, other enteral tube feeds, foods, drinks and oral nutritional supplements) for existing peptide feed users increased significantly from 1654 kcal/day (SD 423) at baseline to 1937 kcal/day (SD 545) with the intervention feed (*p* = 0.034) and remained stable for those patients who were existing polymeric users (1753 kcal/day (SD 583) at baseline vs. 1736 kcal/day (SD 564) with the intervention feed, *p* = 0.991). Total energy intake as a percentage of estimated requirements increased significantly for existing peptide feed users from 87% (SD 24) at baseline to 101% (SD 26) with the intervention feed (*p* = 0.032) and remained stable for existing polymeric feed users (91.5% (SD 22) at baseline to 91.5% (SD 22) with the HEHP intervention feed (*p* = 0.990)). Total protein intake (including the intervention feed, other enteral tube feeds, foods, drinks and oral nutritional supplements) for existing peptide feed users and polymeric feed users increased significantly from 73 g/day (SD 18) and 72 g/day (SD 19) at baseline to 89 g/day (SD 24) and 87 g/day (SD 24) with the intervention feed (*p* = 0.009 and *p* = 0.017, respectively). Similarly, protein intake as a percentage of estimated requirements for existing peptide and polymeric feed users increased significantly from 102% (SD 31) and 98.5% (SD 8) at baseline to 121% (SD 29) and 120% (SD 11) with the intervention feed (*p* = 0.004 and *p* = 0.009, respectively) (see Table 4).

### 3.5. Anthropometry

Weight and BMI were stable between baseline (64 kg (SD 15) and 22.5 kg/m^2^ (SD 5.1)) and with the intervention feed (65 kg (SD 14) and 22.8 kg/m^2^ (SD 5.0), *p* = 0.153 and *p* = 0.209), respectively. This was also the case for both existing peptide and polymeric feed users (*p* > 0.05 for all).

### 3.6. Dietetic Goals

From the dietetic goals set at baseline (as listed in Table 3), 87% (*n* = 13) of patients met all dietetic goals set by their managing dietitian while taking the intervention feed, with a further one patient (pt 9) meeting only one of their goals (maintain nutritional status) but not the other (gastrointestinal tolerance improvement). For the remaining patient (pt 3) who did not meet their dietetic goals, this was a consequence of no gastrointestinal improvement and a slight weight gain. The dietetic goal of improved gastrointestinal tolerance was met by 71% (*n* = 5 of 7) of patients and weight maintenance was met by 83% (*n* = 5 of 6) of patients. For the dietetic goals of nutritional status maintenance (*n* = 4), meeting nutrient requirements (*n* = 2), reintroducing oral diet (*n* = 1), increasing weight (*n* = 1), tolerating increased volume of enteral feed (*n* = 1) and reducing the need for PERT (*n* = 1), these were met by 100% of patients for all goals.

### 3.7. Safety

There were no major safety concerns relating to the intervention feed reported during the study. There was one serious adverse event where inpatient hospitalisation was required following the onset of severe headaches. CT scan and relevant biochemistry measures, however, were normal, yet increased intracranial pressure was diagnosed after lumbar puncture and, consequently, the patient’s clinical team deemed this event not related to the intervention feed. The patient consequently completed the study with no compliance or tolerance issues. There were also three adverse events reported: two patients experienced acute episodes of vomiting and were deemed possibly related to the intervention feed but both patients completed the study with no other compliance or tolerance issues. The other patient had an incidence of reflux due to running out of reflux medication. Consequently, the event was deemed not related to the intervention feed and the patient completed the study with no other compliance or tolerance issues.

## 4. Discussion

The major findings of this pilot study were reduced incidence and intensity of diarrhoea, improved compliance and increased energy and protein intake (via a combination of oral intake and enteral feeds) that better met energy and protein requirements following the use of a high-energy, high-protein, peptide-based, MCT-containing, multi-nutrient enteral tube feed (HEHP) in adult, enterally fed, community patients with complex conditions and impaired gastrointestinal function. The results presented throughout this paper hold important implications for healthcare professionals managing patients at risk of gastrointestinal impairment and represents one of the few studies of its type undertaken in community-based patients.

When gastrointestinal function is impaired (as observed in this patient group), patients often require nutrients to be presented to the intestinal mucosa in a pre-digested form for optimal absorption. It is acknowledged that hydrolysed proteins and more easily digestible fats such as medium-chain triglycerides (MCT) are the best way to supply protein and fats to those with impaired gastrointestinal function as opposed to whole proteins or free amino acids and long-chain fats, respectively [15,16,17,18,33]. The significant improvement in the incidence and intensity of diarrhoea observed in this study adds to the accumulating evidence that provision of enteral tube feeds that contain peptide-based sources of protein and MCT can help mitigate gastrointestinal tolerance issues compared to polymeric equivalents [15,16,17,18]. Furthermore, an improvement in the incidence and intensity of diarrhoea to the intervention feed was also observed compared to previous use of peptide-based enteral tube feeds in this study. For patients who were existing polymeric feed users, most gastrointestinal symptoms were absent and for others remained unchanged between baseline and after receiving the intervention feed. Collectively, there was a tendency for the incidence and intensity of symptoms of constipation, abdominal pain, nausea and bloating to improve during the intervention period (Figure 2), but, likely due to the small number of patients in this pilot study, this did not reach statistical significance, but it may have clinical importance. From a mechanistic standpoint, peptide-based enteral formulas may facilitate an optimum digestive process ultimately leading to an absorptive advantage compared with polymeric-based enteral formulas [13]. Hydrolysed proteins may also improve nitrogen retention, which may, in turn, enhance intestinal microcirculation and thereby improve absorption and gastrointestinal tolerance [34]. Commercially available peptide-based enteral feeds may contain very different blends of peptides which vary in Dalton sizes. The level of free amino acids between formulas also varies. At present, it remains to be fully understood which peptide blends and ranges of peptide sizes are the most advantageous for improving gastrointestinal tolerance and, while speculative, the peptide blend used in this study alongside the predominant MCT fat in the formula could explain the improvements in gastrointestinal tolerance observed here. In addition, the high-energy, high-protein nature of the feed led to significantly reduced total enteral feeding volumes, which may have further contributed to the tolerance of the feed. Importantly, it is highly likely that the improvements observed in patients’ gastrointestinal tolerance influenced the observed improvements in compliance, nutrient intake and dietetic goals, ensuring that not only were nutrient requirements being met more effectively, but also that the nutrients were more likely to be absorbed and used by the body, rather than lost through vomiting or diarrhoea. Nonetheless, further research including greater numbers of patients is encouraged to elucidate the gastrointestinal improvements shown with the use of this high-energy, high-protein, peptide-based enteral tube feed, but as this study is one of a very limited number of studies concerning the use of peptide-based feeds in the community, it adds considerable weight to the safe and effective use of these specialist feeds in the home and care home settings for adults.

The observation of significantly improved compliance with the HEHP intervention feed is an encouraging finding. Furthermore, improved compliance with the intervention feed was also observed compared to previous use of peptide-based enteral tube feeds in this study. Again, no differences in compliance were observed compared to previous use of polymeric enteral tube feeds but this is likely attributed to the small number of patients taking a feed of this nature at baseline. There are many factors proposed to influence patient compliance, but most relate to oral consumption rather than enteral tube feeding [35,36,37]. Although the reasons behind the significantly greater compliance with the intervention feed were not recorded in this pilot study, it is conceivable that improvements in gastrointestinal tolerance, leading to less feeding discomfort or distress, were partly responsible for allowing the full tube feeding regimen to be provided. Furthermore, and although anecdotal, several comments from dietitians and patients throughout this study described less pain during feeding, better motivation to feed, improved mental health and improved energy levels. The lower feeding volume and peptide-blend of the intervention feed may also have contributed to the improved compliance compared to baseline regimens. Compliance is essential when considering clinical outcomes for nutritionally vulnerable populations. Increasing and maintaining adequate compliance can bolster nutritional intake and lower nutritional losses which, over time, may improve body composition and clinical and functional outcomes. Indeed, energy and protein intake not only significantly increased with use of the intervention feed compared to baseline but enabled patients to better achieve estimated energy and protein requirements whilst maintaining weight, although an increase in oral intake was observed in some patients. In this sense, total energy intake was increased to 99% of estimated requirements, which was considered a positive outcome by the recruiting dietitians. Total protein intake (including the intervention feed, other enteral tube feeds, foods, drinks and oral nutritional supplements) was also increased and exceeded 100% of estimated requirements. The increase in protein intake was mostly considered positive by the investigating dietitians and not deemed problematic over the short observation period of this study, but the authors note that there could be more long-term concerns in vulnerable patient groups. During the study, clinical condition improved for several patients while taking the intervention feed, which, in part, may explain the observed increases in energy and protein intake, particularly from oral sources. It is important to note, however, that several bodies have recently updated calculations for estimating energy and protein requirements [38]. These were not available at the time of the study and are now based on data from clinical settings. If using these updated calculations, estimated protein requirements are higher and total protein intake as a percentage of estimated requirements would be lower at baseline and with the intervention feed (i.e., closer to 100% at the end of the intervention period). The observed increases in nutritional intake in this study are likely in part due to the improved gastrointestinal tolerance and compliance with the intervention feed, which may have been facilitated by the lower feeding volume and peptide-blend of the intervention feed. Furthermore, the increase in oral food intake for some patients while taking the intervention feed may also explain the observed increases in nutritional intake. Similar increases in energy and protein intake have led to clinical (quality of life) and functional (hand grip strength) improvements with a polymeric enteral feed containing similar levels of energy and protein: 1.5 kcal/mL and 7.5 g protein/100 mL (Nutrison Protein Plus Energy^®^, Nutricia Ltd.) [32]. Whether the findings of improved gastrointestinal tolerance, compliance and improved nutritional intake in this pilot study would translate to improvements in quality of life and hand grip strength should be determined in future research. It would also be interesting to assess whether the use of the intervention feed would be as effective in a critical care or acute setting.

While the patients that completed this pilot study were an extremely heterogeneous group, due to the fact that all patients recruited were adult, community-based, enterally fed patients who required a peptide-based feed, it could be argued they were homogenous to some extent and reflect the patients who require this type of feed in clinical practice. More often, patients presented with different and multiple diagnoses. Medical conditions were therefore complex and, prior to recruitment, patient experiences and clinical journeys varied greatly. The reason for recruitment to the pilot study therefore varied and, consequently, dietetic goals differed across patients. As an adjunct to the wider aim of this pilot study, the managing/recruiting dietitians reported that 87% (*n* = 13) of patients met all dietetic goals set. This would suggest that the intervention feed helped patients to successfully achieve dietetic goals relevant to their clinical care in the short timeframe of this study, including goals of improved gastrointestinal tolerance, weight management and improved nutritional intake.

These findings are not without limitation. Firstly, as this was a pilot study, caution should be taken when extrapolating the results, as the findings are constrained to a relatively small number of patients over a short observational period. It would be advantageous to follow on from this body of work by expanding recruitment in community patients and broadening the assessment of outcome measures where possible (e.g., nutritional status, functional measures and clinical outcomes) over more frequent timepoints. However, access to this complex, vulnerable patient group can be challenging for the purposes of clinical trials. Similarly, it would also be of value to investigate the benefits of such a feed in acutely ill patient groups, including those from a critical care setting, in adequately powered randomised controlled trials where feasible. Secondly, dietary intake data were obtained via 24 h dietary recall, which is an effective method for providing detailed intake data on specific days with relatively small respondent burden compared to other methods (which is important when working with patients with complex conditions); however, there is potential for recall bias [39]. Nonetheless, 67% of patients (*n* = 10 of 15) in this study were NBM at recruitment and others had relatively low oral intakes, therefore increasing the confidence in our dietary intake data, which was recorded from tube feeding regimens. Thirdly, the patient group was heterogeneous in nature and therefore different to the previous disease-specific trials published in this area, but it represents a realistic cross-section of patients using peptide-based feeds in clinical practice. Finally, an assessment of the differences between the peptide-blends used in the different tube feeds may elicit a possible mechanism for the improved tolerance compared to other peptide-based feeds observed in this trial, and this should be an avenue for future research. To strengthen outputs, any further studies should consider objective assessments of absorption (e.g., coefficient of fat absorption/coefficient of nitrogen absorption). It would also be prudent if future research considered more long-term observation periods to identify whether intake of the intervention feed and other peptide-based feeds impacts body composition and prescription of condition-specific medications.

## 5. Conclusions

The results of this pilot study demonstrate that a high-energy, high-protein, peptide-based, MCT-containing enteral tube feed maintains gastrointestinal tolerance and improves regimen compliance, leading to improvements in energy and protein intake to meet nutritional goals in complex, community-based, adult, tube-fed patients and may present better GI symptom relief compared to other commercially available, peptide-based enteral tube feeds. This study also demonstrates that the HEHP intervention feed is safe and effective in meeting dietetic goals, although further research is required to assess the benefits in the longer term to patient outcomes.

## Figures and Tables

**Table 1:** Nutritional composition of the HEHP (high-energy, high-protein) intervention feed *.

	Unit	Per 100 mL
Energy	Kcal (kJ)	150 (631)
Protein (whey hydrolysate)	g	7.5
Nitrogen	g	1.2
Carbohydrate	g	18.7
Fat (of which MCT)	g	5.0 (3.0)
Fibre	g	<0.5
Sodium	mg (mmol)	197 (8.56)
Potassium	mg (mmol)	340 (8.70)
Chloride	mg (mmol)	60.0 (1.69)
Calcium	mg (mmol)	97.2 (2.43)
Phosphorus	mg (mmol)	97.0 (3.13)
Magnesium	mg (mmol)	30.0 (1.23)
Osmolarity	mOsmol/L	445
Osmolality	mOsmol/kg water	580
	pH	7.7

* The intervention feed was a nutritionally complete, ready-to-use, high-energy (1.5 kcal/mL), high-protein (7.5 g protein/100 mL), peptide-based, MCT (medium-chain triglycerides)-containing enteral tube feed, nutritionally complete in 1000 mL.

**Table 2:** Baseline patient characteristics: age (years), weight (kg), BMI (kg/m^2^), primary diagnosis and indication for HEN (Home Enteral Nutrition).

Patient ID	Age (Years)	Weight (kg)	BMI (kg/m^2^)	Primary Diagnosis	Indication for HEN
1	19	47	19.1	Idiopathic gastroparesis	Disease-related malnutrition
2	48	67	27.2	Severe acute necrotising pancreatitis	Delayed gastric emptying
3	22	67.7	23.4	Cerebral palsy and epilepsy	Nil by mouth
4	20	40.6	16.1	Cerebral palsy and epilepsy	Unable to eat enough food orally due to dysphagia
5	52	70.5	30.5	Severe acute pancreatitis	Delayed gastric emptying
6	38	50.6	18.8	Global slow bowel transit	Unable to maintain weight via oral diet due to vomiting
7	38	57.5	19.4	Antiphospholipid syndrome	Gastric bypass leading to malabsorption
8	43	61.1	17.9	Idiopathic gastroparesis	Disease-related malnutrition
9	58	96.8	33.5	Roux-en-Y anastomosis	Gastric bypass leading to poor tolerance of solid foods and poor nutritional status
10	60	72.3	22.4	Severe acute pancreatitis	Delayed gastric emptying
11	36	85	24.7	Motor neurone disease	Nil by mouth
12	26	58	20.1	Gastroparesis	Gastroparesis. Unable to eat enough food orally
13	68	47.4	15.7	Pancreaticoduodenectomy	Partial gastrectomy and pancreatitis. Long standing poor nutritional status
14	66	73.6	24.7	Haemorrhagic stroke	Dysphagia following haemorrhagic stroke
15	36	66.7	23.9	Gastroparesis	To supplement oral intake and maintain weight
16	49	77.5	23.9	Pancreaticoduodenectomy	Delayed gastric emptying
17	72	96.5	30.5	Severe acute pancreatitis	Duodenal outlet obstruction
18	48	47.1	18.9	Cerebral palsy and epilepsy	High risk of aspiration on oral intake
19	69	71	23.5	Mandibular squamous cell carcinoma	Dysphagia secondary to radiotherapy
20	24	49.5	18.6	Congenital hydrocephalus, cerebral palsy and epilepsy	Dysphagia—nil by mouth
Mean	44.6	65.2	22.6		
SD	17.2	16.0	4.9		

**Table 3:** Baseline patient characteristics: energy requirement (kcal/day), protein requirement (g/day), feeding route, feeding method, dietetic goal and days on study.

Patient ID	Energy Requirement (kcal/day)	Protein Requirement (g/day)	Feeding Route	Feeding Method	Dietetic Goal	Days on Study
1	2043	56	NJ	Continuous	1	14 ^¥^
2	2150	105	NJ	Continuous	1, 2	28
3	2279	72	LPBG	Bolus	1, 3	28
4	1905	52	LPBG	Continuous	1, 3	28
5	2115	106	NJ	Continuous	4	28
6	1780	54	PEJ	Continuous	5	28
7	1973	86	PEJ	Continuous	1	28
8	1637	65	SJ	Continuous	3, 6	25 ^≠^
9	1637	77	SJ	Continuous	3, 4	21 ^†^
10	2024	90	NJ	Continuous	2, 4	28
11	1397	69	PEG	Bolus	6	28
12	1647	58	PEJ	Continuous	3, 7	28
13	1806	62	LPBG	Continuous	5, 8	28
14	2455	92	PEG	Continuous	1, 3	28
15	1868	67	SJ	Continuous	3, 4	28
16	2287	116	PEG	Continuous	1	3
17	2188	87	NJ	Continuous	1	13
18	1167	51	PEG	Continuous	9	3
19	2086	75	NJ	Continuous	1, 10	10
20	1050	42	PEG	Continuous	4	4
Mean	1874.7	74.1				
SD	369.6	20.5				

Dietetic goals: 1 = weight maintenance; 2 = reintroduce oral diet; 3 = gastrointestinal tolerance improvement; 4 = maintain nutritional status; 5 = increase weight; 6 = meet nutrient requirements; 7 = tolerate increased volume of feed; 8 = reduce need for pancreatic enzyme replacement therapy; 9 = reduce episodes of vomiting; 10 = maintain gastrointestinal tolerance. Feeding route: NJ = nasojejunal; LPBG = low profile balloon gastrostomy; PEJ = percutaneous endoscopic jejunostomy; PEG = percutaneous endoscopic gastrostomy; SJ = surgical jejunostomy. ^¥^ patient only completed 14 days due to experiencing increased constipation, but otherwise gastrointestinal tolerance was good and reported less pain during feeding. ^≠^ patient completed 25 days due to timing of end of study review. ^†^ patient completed 21 days due to significant clinical improvement, permitting reintroduction of oral intake, weaning off enteral feed and consequently enteral tube removal.

**Table 4:** Mean (SD) total energy and protein intake, percentage of estimated requirements and delivered amounts of enteral feeding at baseline and end of intervention (HEHP).

	Baseline	HEHP	*p* Value
**All patients (*n* = 15)**			
Total energy intake (kcal/day) ^1^	1675 (449)	1884 (537)	0.039
Total energy intake as % of estimated requirements (%) ^2^	88 (30)	99 (25)	0.038
Total protein intake (g/day) ^1^	73 (17)	89 (23)	0.001
Total protein intake as % of estimated requirements (%) ^2^	101 (23)	121 (25)	<0.001
Total enteral feed volume delivered (mL)	1068 (507)	871 (396)	0.233
Total energy intake from enteral feeds only (kcal/day)	1421 (583)	1468 (544)	0.097
Total protein intake from enteral feeds only (kcal/day)	62 (22)	72 (25)	<0.001
**Existing peptide-based feed users (*n* = 11)**			
Total energy intake (kcal/day) ^1^	1654 (423)	1937 (545)	0.034
Total energy intake as % of estimated requirements (%) ^2^	87 (24)	101 (26)	0.032
Total protein intake (g/day) ^1^	73 (18)	89 (24)	0.009
Total protein intake as % of estimated requirements (%) ^2^	102 (31)	121 (29)	0.004
Total enteral feed volume delivered (ml)	980 (453)	824 (400)	0.050
Total energy intake from enteral feeds only (kcal/day)	1399 (546)	1441 (523)	<0.001
Total protein intake from enteral feeds only (kcal/day)	62 (22)	71 (240	<0.001
**Existing polymeric feed users (*n* = 4)**			
Total energy intake (kcal/day) ^1^	1753 (583)	1736 (564)	0.991
Total energy intake as % of estimated requirements (%) ^2^	91.5 (22)	91.5 (22)	0.990
Total protein intake (g/day) ^1^	72 (19)	87 (24)	0.017
Total protein intake as % of estimated requirements (%) ^2^	98.5 (8)	120 (11)	0.009
Total enteral feed volume delivered (mL)	1000 (408)	1000 (408)	1.000
Total energy intake from enteral feeds only (kcal/day)	1478 (790)	1540 (679)	0.005
Total protein intake from enteral feeds only (kcal/day)	60 (25)	76 (31)	0.002

^1^ At baseline, total energy intake included the patients’ primary feed, other enteral tube feeds, foods, drinks and oral nutritional supplements. At the end of the study, total energy intake included the intervention feed, other enteral tube feeds, foods, drinks and oral nutritional supplements. ^2^ After actual intakes were computed, they were compared against dietitians’ calculated estimated energy and protein requirements and percentage achievements were calculated.
